# Tambjamines and Prodiginines: Biocidal Activity against *Trypanosoma cruzi*

**DOI:** 10.3390/pharmaceutics13050705

**Published:** 2021-05-12

**Authors:** Rocío Herráez, Roberto Quesada, Norma Dahdah, Miguel Viñas, Teresa Vinuesa

**Affiliations:** 1 Department of Pathology and Experimental Therapeutics, Medical School, University of Barcelona, 08021 Barcelona, Spain; rherraez@ub.edu (R.H.); mvinyas@ub.edu (M.V.); 2 Departamento de Química, Universidad de Burgos, 09001 Burgos, Spain; rquesada@ubu.es; 3 Departament de Ciències Fisiològiques, Medical School, University of Barcelona, 08907 Barcelona, Spain; dahdahnorma@gmail.com

**Keywords:** *Trypanosoma cruzi*, tambjamines, prodigiosin, obatoclax, anti-chagasic agents, cytotoxicity, targets, mitochondria

## Abstract

The aim of this work was to explore new therapeutic options against Chagas disease by the in vitro analysis of the biocidal activities of several tambjamine and prodiginine derivatives, against the *Trypanosoma cruzi* CLB strain (DTU TcVI). The compounds were initially screened against epimastigotes. The five more active compounds were assayed in intracellular forms. The tambjamine MM3 and both synthetic and natural prodigiosins displayed the highest trypanocidal profiles, with IC_50_ values of 4.52, 0.46, and 0.54 µM for epimastigotes and 1.9, 0.57, and 0.1 µM for trypomastigotes/amastigotes, respectively. Moreover, the combination treatment of these molecules with benznidazole showed no synergism. Finally, oxygen consumption inhibition determinations performed using high-resolution respirometry, revealed a potent effect of prodigiosin on parasite respiration (73% of inhibition at ½ IC_50_), suggesting that its mode of action involves the mitochondria. Moreover, its promising selectivity index (50) pointed out an interesting trypanocidal potential and highlighted the value of prodigiosin as a new candidate to fight Chagas disease.

## 1. Introduction

Chagas disease, also known as American trypanosomiasis, is caused by the protozoan *Trypanosoma cruzi*. This neglected infectious disease affects 6 to 7 million people worldwide and is endemic in 21 countries of Latin America [1,2]. Nowadays, due to migratory flows, Chagas disease has spread to non-endemic countries such as Spain, Canada, Australia, Japan, and North America, becoming a relevant public health challenge [3,4].

Chagas disease has two clinical phases. In the acute phase, the parasite disseminates and can be detected by direct bloodstream examination [5]. In most cases, it courses without symptoms and if there are any symptoms, they are usually mild or nonspecific. The acute phase usually lasts up to 2 months and resolves spontaneously. However, in 5–10% of infected subjects, the disease may become fatal because of heart failure or meningoencephalitis [6,7]. After the acute phase, the disease enters the chronic phase, which can adopt two forms. In about 60–70% of cases, the disease remains in the indeterminate form, which may last 10–30 years or for the lifetime of the patient. This form is silent and free of symptoms. In the remaining cases (30–40% of individuals), the patients develop a chronic form of the disease, which mostly involves severe cardiac, digestive, and neurological disorders [8,9,10].

For more than 40 years, only two drugs have been available for the treatment of Chagas disease: nifurtimox and benznidazole. Both have severe side effects that lead to treatment discontinuation in 15–20% of cases [11,12,13,14]. This, coupled with their limited efficacy in the chronic phase of the disease [15] and the occurrence of naturally resistant strains [16], reinforces the need to identify new compounds to fight Chagas disease. Combination therapy has emerged as an alternative in the treatment of Chagas disease. Synergistic drug combinations could improve current regimens, allowing doses to be reduced and consequently diminishing side effects [17,18].

In our search for new trypanocidal drugs, we focused on tambjamines, a family of naturally occurring compounds isolated from bacteria and marine invertebrates such as ascidians, bryozoans, and nudibranchs [19]. Tambjamines are alkaloids structurally related to prodiginines, sharing with them the 4-methoxy-2,2′-bipyrrole core and also a wide spectrum of useful biological properties that include antimicrobial, antifungal, antimalarial, and antitumor activities [20,21,22].

Although the mode of action of tambjamines has not been elucidated yet, it seems to be closely linked to its ability to act as anion transporters, a feature that is also shared by prodiginines [20,23]. Furthermore, the mitochondria of *T. cruzi* have been regarded as a potential target, since alterations in the mitochondria have catastrophic effects on respiration and, subsequently, on the survival of the parasite [24].

Surprisingly, to our knowledge, there are no studies on the use of tambjamines as antitrypanosomal agents. However, it has been postulated that these compounds exert a remarkable effect on other protozoans and have been proposed as antimalarial agents. Kancharla et al. [25] described the successful resolution of a *Plasmodium yoelii* infection in a murine model through treatment with tambjamines. Specifically, the synthetic tambjamine KAR425 had potent in vivo activity against the *P. yoelii* infection, showing no signs of cell toxicity.

Our group recently studied prodigiosin of bacterial origin, reporting its remarkable activity against the epimastigotes of *T. cruzi* (CL strain, clone B5), with very low IC_50_ values (up to 0.8 µM), compared to benznidazole (18 µM). Additionally, we explored the effects of this pigment on the parasite cell surface by atomic force microscopy, which revealed relevant morphological alterations in the size, shape, and roughness [26]. Based on these encouraging preliminary results, we decided to continue exploring the trypanocidal effect of prodigiosin and related molecules against the intracellular forms of *T. cruzi* and investigate the targets presumably involved.

The aims of the present work were to (1) determine the in vitro trypanocidal activity of natural and synthetic prodiginines as well as several tambjamine derivatives against the epimastigotes and trypomastigotes/amastigotes of the parasite, (2) evaluate the toxicity of these compounds in two cultured mammalian cell lines, (3) determine their biological activity in combination with the standard drug benznidazole, and (4) explore their mode of action.

## 2. Materials and Methods

### 2.1. Drugs

**Figure 1:**
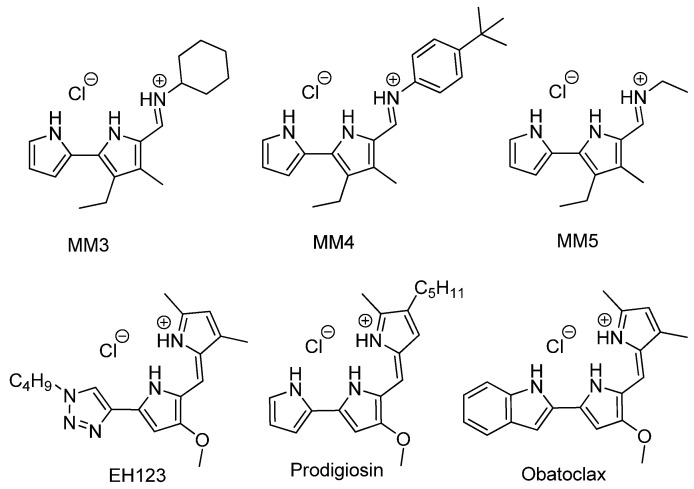
Chemical structure of the studied compounds.

Benznidazole (BNZ, Laboratorios ELEA, Buenos Aires, Argentina) was used as the reference drug. Tambjamine analogs, Obatoclax, and synthetic prodigiosin (prepared as a hydrochloride salt) were synthesized as reported elsewhere [27,28,29]. Natural prodigiosin was obtained as previously described [26]. In natural extracts, it is feasible that related family compounds may also be present and protonation state may vary with both protonated and neutral forms coexisting depending on the pH level. Figure 1 shows the chemical structures of the synthesized molecules.

### 2.2. Cultures

#### 2.2.1. Cell Cultures

Mouse L-929 fibroblasts (NCTC clone 929, ECACC 88102702) were used for both drug toxicity and infection assays. Cell line was obtained from Dr. Concepció Soler, Dept. Pathology & Experimental therapeutics, Faculty of Medicine and Health Sciences, University of Barcelona, Spain. Human hepatocarcinoma Hep G2 cells (American Type Culture Collection (ATCC HB-8065)) were employed only in the drug toxicity assays and were obtained from Dr. Neus Agell, Cell Biology Research Group, Faculty of Medicine and Health Sciences, University of Barcelona, Spain.

Cell lines were maintained in MEM and RPMI 1640 media (Biochrom AG, Berlin, Germany), respectively, supplemented with 10% fetal bovine serum (FBS; Gibco, Life Technologies, Carlsbad, NY, USA) and 100 µg/mL of streptomycin + 100 U/mL of penicillin G (Sigma-Aldrich, St. Louis, MO, USA). Cells were kept at 37 °C in an atmosphere of 5% CO_2_.

#### 2.2.2. Parasite Cultures

Epimastigotes of *T. cruzi* (CL strain, clone B5) were cultured axenically at 28 °C in liver infusion tryptose medium (LIT; Difco BD, MD; Pronadisa, Madrid, Spain), supplemented with 10% FBS and antibiotics. The CL-B5 strain was stably transfected with the *Escherichia coli* β-galactosidase gene (lacZ) and routinely maintained in exponential growth by weekly passages. Trypomastigotes of the CL-B5 strain were obtained as previously reported [30].

### 2.3. Biological Assays

#### 2.3.1. Epimastigote Susceptibility Assay (Monotherapy)

The screening assays were performed in 96-well microplates (WWR Int LLC) containing serially diluted concentrations of each compound and seeded with 2.5 × 10^5^ epimastigotes/mL harvested during the exponential growth phase. Growth, medium, and drug blanks were used in each assay as controls. An internal control of the reference drug, benznidazole, was also included. After 72 h at 28 °C, 50 µL of the substrate chlorophenol red-β-D-galactopyranoside (CPRG; Roche) diluted in 0.9% Triton X-100 solution (EMD, Darmstadt, Germany) were added at a final concentration of 200 µM. Then, after 3 h at 37 °C, measurements were performed in a scanning multiwell spectrophotometer (ELISA Multiskan Reader, BioTek Instruments, Inc., Winooski, VT, USA) at 595 nm. Each concentration was tested in triplicate and the experiments were performed three times independently. The efficacy of the compounds was calculated by determining the percentage of epimastigote growth (% EG value) as follows:

(1) $$\%\;\mathrm{Epimastigote}\;\mathrm{growth}=\frac{\left(\mathrm{AE}-\mathrm{AEB}\right)}{\left(\mathrm{AC}-\mathrm{ACB}\right)}\times 100$$

where AE is the absorbance of the experimental group, AEB the absorbance of the drug blank, AC the absorbance of the control group, and ACB the absorbance of the culture medium blank. The IC_50_ values (the drug concentration that eliminates 50% of the parasites) were determined for each compound using the GraphPad Prism software (Version 5.0.0 for Windows, GraphPad Software, San Diego, CA, USA).

Molecules showing activity against epimastigotes similar to or greater than that of benznidazole were then evaluated against the intracellular and trypomastigote forms of the parasite.

#### 2.3.2. Trypomastigote/Amastigote Susceptibility Assay

This assay was performed in 96-well microplates. Briefly, 4 × 10^3^ L-929 fibroblasts were seeded in 80 µl of MEM containing 10% FBS and antibiotics. The plate was placed overnight at 37 °C in a 5% CO_2_ humidified incubator to allow cell adherence. Afterward, the cells were infected with 20 µL of trypomastigotes (40,000 trypomastigotes/well (4000 mammalian cells)) diluted in the same medium. The cell:parasite ratio was 1:10. Plates were incubated for 2 h at 33 °C and 5% CO_2_. The medium was then discarded to dispose of nonpenetrated parasites and replaced with 200 µL of MEM without phenol red supplemented with FBS and antibiotics. After 48 h of incubation, the content of the wells was replaced with a fresh medium containing the dilutions of the compounds. The plates were returned to the incubator for an additional 96 h under the same conditions. Wells containing drug blanks, medium, fibroblasts, and untreated infected cells were included in all the plates as controls. An internal control of the reference drug (benznidazole) was also included. Next, 50 µl of substrate CPRG (diluted in 3% Triton X-100 solution) were added at a final concentration of 400 µM. After 18–20 h of incubation at 37 °C, absorbance readings were taken at 595 nm using a scanning multiwell spectrophotometer. All the assays were performed in triplicate and the experiments were run three times in different weeks. The results are expressed as the percentage of trypomastigote/amastigote growth inhibition (% T/A GI value) as follows:

(2) $$\%\mathrm{Trypomastigote}/\mathrm{Amastigote}\;\mathrm{growth}\;\mathrm{inhibition}=100-\frac{\left(\mathrm{AE}-\mathrm{AEB}\right)}{\left(\mathrm{AC}-\mathrm{ACB}\right)\;}\times 100$$

where AE is the absorbance of the experimental group, AEB the absorbance of the compound blank, AC the absorbance of the control group, and ACB the absorbance of the culture medium blank. The IC_50_ values (the drug concentration that eliminates 50% of the parasites) were determined for each compound using the GraphPad Prism software.

#### 2.3.3. Epimastigote Susceptibility Assay (Combined Therapy)

To assess the anti-epimastigote effect of the compounds in combination with benznidazole, fixed concentrations of each compound were added to a 96-well microplate previously filled with serial dilutions of benznidazole (0.06–128 µM). These fixed concentrations were calculated based on the IC_50_ values previously obtained for each drug and corresponded to 4x IC_50_, 2x IC_50_, IC_50_, 0.75x IC_50_, 0.5x IC_50_, 0.25x IC_50_. Each well was inoculated with 100 µL of the epimastigotes (2.5 × 10^5/mL^) obtained from the exponential growth phase and the plates were incubated for 72 h at 28 °C. Drug blanks, medium, and growth wells were included in each assay as controls. Afterward, 50 µL of CPRG diluted in 0.9% Triton X-100 solution were added at a final concentration of 200 µM and the plates were returned to the incubator for another 3 h at 37 °C. After incubation, absorbance was read at 595 nm and the data were analyzed. All experiments were repeated at least two times independently and each combination was tested in duplicate.

The results are expressed as the IC_50_ values obtained with each drug combination. To evaluate the interaction of benznidazole with these compounds, fractional inhibitory concentration (FIC) values were calculated as follows:

FIC of compound A (FIC A) = IC_50_ of A in combination/IC_50_ of A alone; while FIC of compound B (FIC B) = IC_50_ of B in combination/IC_50_ of B alone.

Finally, the FIC index (FICi) was calculated as the sum of FICs using the following formula:

FICi=FIC A+FIC B

FICi values were interpreted according to the criteria established by Simoes-Silva et al. [31]: FICi ≤ 0.5 indicates synergism; FICi > 0.5 and ≤ 4 indicates an additive interaction (no interaction); and FICi > 4 indicates antagonism.

#### 2.3.4. Cytotoxicity Assays

Cells were seeded by trypsinization in 96-well microplates (L-929: 4 × 10^3^ cells/well; Hep G2: 1.5 × 10^4^ cells/well) and incubated for 24 h at 37 °C to allow cell attachment. On the following day, the medium was discarded and replaced with a fresh medium containing the different concentrations of the drugs. Hep G2 cells were treated with concentrations of 100, 10, and 1 µM of the different drugs, while L-929 cells were treated with a wider range of concentrations in order to calculate the IC_50_ value (the drug concentration that inhibits 50% of cell growth). Medium, growth, and drug blanks were used in each test as controls. Plates were incubated again for another 24 h. After treatment, 20 µL of resazurin diluted in 1X phosphate-buffered saline (PBS; Sigma Aldrich) was added at a final concentration of 70 µM. The plates were incubated for another 24 h, after which fluorescence was measured in a microplate reader (FLUOstar OPTIMA, BMG LABTECH GmbH, Ortenberg, Germany) at an excitation wavelength of 530 nm and an emission wavelength of 590 nm. Each concentration was tested in triplicate and the experiments were repeated three times independently. Results indicated the percentage of toxicity (% T value).

(3) $$\%\;\mathrm{Toxicity}=100-\frac{\left(\mathrm{AE}-\mathrm{AEB}\right)}{\left(\mathrm{AC}-\mathrm{ACB}\right)}\times 100$$

where AE is the absorbance of the experimental group, AEB the absorbance of the drug blank, AC the absorbance of the untreated cells, and ACB the absorbance of the culture medium blank. For the L-929 fibroblasts, the IC_50_ values (the drug concentration that inhibits 50% of cell growth) were calculated for each compound using the GraphPad Prism software.

### 2.4. Oxygen Consumption Inhibition Analysis

Intact epimastigotes were collected by centrifugation, washed twice in PBS, and resuspended with respiration buffer [32] containing 125 mM sucrose, 65 mM KCL, 10 mM Tris-HCl (pH 7.2), 1 mM Mg_2_Cl, and 2.5 mM K_2_PO_4_. They were then added to a final concentration of 25 × 10^6^ cells/mL.

To evaluate the effect of the different drugs on mitochondrial function, we measured mitochondrial respiration in the cells before and after the addition of increasing volumes of the compounds. Each compound was added in titrations until reaching concentrations of ½ and ¾ IC_50_. Finally, antimycin A (AA) was added to determine the residual oxygen consumption (ROX) through the inhibition of complex III of the electron transport chain. The ROX value was subtracted from the O_2_ flux as a baseline for all the respiratory states [33].

Mitochondrial respiration was measured by high-resolution respirometry (OROBOROS Oxygraph-2k; OROBOROS Instruments, Innsbruck, Austria). Data were analyzed by using the Software DatLab Online Data Acquisition and Analysis (OROBOROS Instruments, Innsbruck, Austria). All respirometry assays were performed at a constant temperature of 28 °C, with the volume of each chamber being 2 mL. The oxygen flux values are expressed relative to the cell concentration per second (pmol/sec/10^6^ cells). The effect of each compound on mitochondrial function is shown as the percentage of inhibition of oxygen flux.

### 2.5. Transmembrane Anion Transport Experiments

The transmembrane anion transport activity of the compounds was explored in phospholipid (1-palmitoyl-2-oleoyl-sn-glycero-3-phosphocholine (POPC)) vesicles. Chloride efflux from chloride loaded vesicles was monitored using a chloride selective electrode. A solution of the studied compound in DMSO, usually 5 µL to avoid the influence of the organic solvent during the experiments, was added, and once the experiment was finished, a surfactant (Triton-X, 10% dispersion in water, 20 µL) was used to lyse the vesicles and release all the encapsulated chloride. This value was taken as 100% release and used as such. The experiments were repeated using different concentrations of the studied compounds and normalized chloride efflux at 300 against the concentration of compound plotted. The data were then fitted with the Hill equation and an EC50 parameter, representing the concentration of compound eliciting 50% of chloride release was calculated. Assays in which the external medium was nitrate or bicarbonate were performed.

## 3. Results

### 3.1. Epimastigote Susceptibility Assay (Monotherapy)

**Figure 2:**
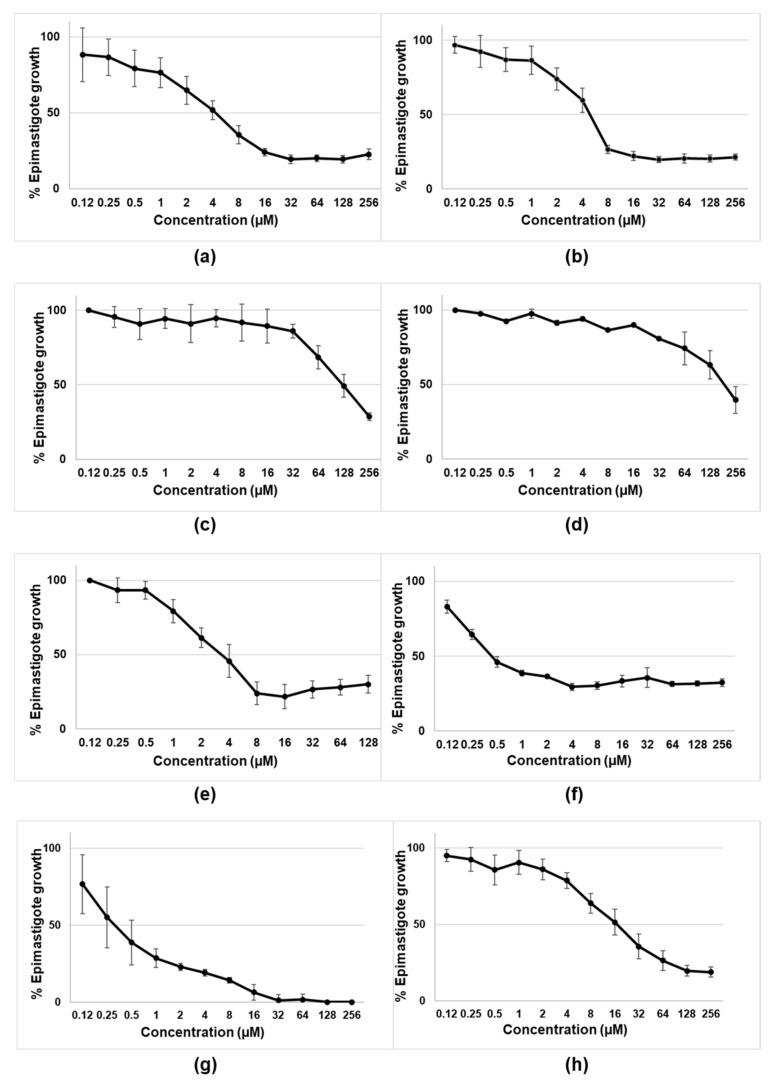
Biological activity of tambjamines: (**a**) MM3; (**b**) MM4; (**c**) MM5; (**d**) EH123; (**e**) Obatoclax; (**f**,**g**) synthetic and natural prodigiosins, respectively; and (**h**) benznidazole against epimastigotes of *T.cruzi* (CL-B5 strain). Results are expressed as percentages of epimastigote growth with the corresponding standard deviations. MM3, MM4, MM5, and EH123 are tambjamines. For tambjamines, prodigiosins and benznidazole (**a**–**d**,**f**–**h**), the tested concentrations ranged from 0.12 µM to 256 µM. For Obatoclax (**e**), the tested concentrations ranged from 0.12 µM to 128 µM.

The initial experiments were designed to assess the activity of tambjamines, Obatoclax, and natural and synthetic prodigiosins on the epimastigotes of *T. cruzi* (CLB strain-DTU VI). As shown in Figure 2 and Table 1, the most active compounds were the tambjamines derivatives MM3 and MM4, Obatoclax, and both the natural and synthetic prodigiosins. Among them, natural and synthetic prodigiosins displayed the best trypanocidal profiles, with IC_50_ of 0.54 µM and 0.46 µM, respectively. On the contrary, MM5 and EH123 did not show detectable activity against the extracellular and replicative forms of *T. cruzi*.

The compounds with the highest activities from the preliminary drug screening assay were selected for further analyses with the trypomastigote/amastigote forms of *T. cruzi*.

The molecules that were found to be the most active against the epimastigotes (MM3, Obatoclax, and prodigiosins) were then evaluated against the intracellular and trypomastigote forms of *T. cruzi* along with the reference drug benznidazole.

### 3.2. Trypomastigote/Amastigote Susceptibility Assay

Table 2 shows the percentages of growth inhibition by the selected molecules at the maximum concentration assayed (16 µM). At this concentration, all the compounds inhibited trypomastigote/amastigote growth more than benznidazole. Both synthetic and natural prodigiosins had the lowest IC_50_ values (0.57 and 0.1 µM, respectively).

### 3.3. Drug Synergism Assays

For the combined therapy assays, FICi values suggested that none of the drug combinations assayed showed synergism. The FICi values corresponding to the combination of benznidazole and any one of the other compounds at a concentration corresponding to 0.25x IC_50_ (the lowest proportion of all those tested) corroborated the absence of synergy at these concentrations (Table 3).

### 3.4. Cytotoxicity

The percentages of toxicity at the highest drug concentration assayed (16 µM) in a mammalian cell line (L-929 fibroblasts) are shown. To calculate the IC_50_ of EH123 and benznidazole, cytotoxicity was determined over a broader range of concentrations than the studied molecules, because of their low level of toxicity (Table 4).

To assess toxicity values, experiments were also conducted in human hepatocarcinoma cells (Table 5). Similar toxicity values were obtained, with high percentages of toxicity at the drug concentrations of 100 and 10 µM. All the compounds assayed showed acceptable toxicity only at 1 µM, with the cytotoxicity values always lower than 20%. Tambjamines MM3 and MM4 showed the highest IC_50_ values (46.01 and 52.54 µM, respectively).

### 3.5. Selectivity Index (SI)

Despite all the studied compounds being less toxic to fibroblasts than to protozoans, all the values were in a moderate range and always much lower than that of benznidazole (Table 6).

### 3.6. Oxygen Consumption Inhibition Analysis

In the epimastigotes of *T. cruzi*, prodigiosins showed the highest percentage of oxygen consumption inhibition, with the natural prodigiosin eliciting an inhibition of 73% at ½ IC_50_ and the synthetic prodigiosin causing inhibition of 13.58% and 35.18% at ½ IC_50_ and ¾ IC_50_, respectively (Table 7). The tambjamine MM3 also appeared to be effective, with values close to 50% at the concentrations assayed (½ and ¾ IC_50_). Conversely, MM4 and Obatoclax showed little or no effect on respiratory inhibition.

### 3.7. Transmembrane Anion Transport Experiments

Prodigiosin was found to be the most active anionophore, whereas MM5 display very limited activity, and no EC50 parameter could be calculated because this compound did not elicit enough chloride efflux (Table S1).

## 4. Discussion

In this work, we evaluated the biological activity of several tambjamines, Obatoclax, and natural and synthetic prodiginines against *T. cruzi* (CLB strain). The two synthetic tambjamine derivatives (MM3 and MM4) displayed trypanocidal capability against both the extracellular and intracellular forms of *T. cruzi.*

To our knowledge, this is the first report on the trypanocidal effect of tambjamines, although their usefulness has been explored mainly as antimalarial agents [25]. By contrast, derivatives MM5 and EH123 did not show detectable levels of trypanocidal activity and were subsequently discarded for the rest of the experiments. Obatoclax displayed remarkable trypanocidal activity against the epimastigotes of *T. cruzi*; however, its effectiveness against the intracellular forms of *T. cruzi* was slightly lower than that of the other molecules assayed. This is consistent with the results recently reported by Ehrenkaufer et al., who demonstrated that Obatoclax had remarkable activities against *Entamoeba*, *Giardia*, and *Trypanosoma brucei*, with IC_50_ values of 0.5, 0.9, and 0.04 µM, respectively [34]. Here, we provide the effectiveness data of this compound in *T. cruzi*. The observed results correlate well with the ability of these compounds to function as transmembrane anion carriers. Compounds MM3, MM4, and MM5 are synthetic tambjamines bearing different substituents in the enamine moiety. The presence of an ethyl group in MM5 resulted in the lack of trypanocidal activity, as opposed to MM3 and MM4. These two compounds are equipped with larger substituents (cyclohexyl and -4-tertbutylphenyl, respectively) resulting in more lipophilic derivatives better suited to facilitate anion transport. The replacement of a pyrrole heterocycle characteristic of both prodigiosin and obatoclax by a 1,2,3-triazole in the case of EH123 also impact negatively in the ability of this latter compound to interact and transport anions, resulting again in a marked decline in the activity of EH123 as a trypanocidal agent.

It has been pointed out that the mode of action of Obatoclax on cancer cells is mainly through its proapoptotic activity. The drug has the ability to inhibit the Bcl-2 family of proteins. However, trypanosomatids, as unicellular protists, lack apparent Bcl-2 homologs. Therefore, they should be, in principle, resistant to Obatoclax unless the mode of action of Obatoclax involves some more targets than the inhibition of this apoptotic regulator. Ehrenkaufer et al. [34] reported that other Bcl-2 inhibitors such as venetoclax, navitoclax, A-1331852, A-1210477, and S63845 do not have an effect on the viability of different parasites, subsequently suggesting that a different mechanism of action should be investigated. Their results clearly indicated that Obatoclax most likely kills the parasites through a BCL-2-independent mechanism. Our results confirmed the potent trypanocidal activity of prodigiosin, which has been previously demonstrated by our group and others [26,35], confirming its effectiveness against the intracellular forms of *T. cruzi*.

All the compounds assayed, except for EH123, showed significant toxicity, with all of them being more toxic than benznidazole. EH123 showed low toxicity and low antiparasitic activity. Obatoclax and prodigiosins were particularly toxic, showing nonselective cytotoxic behavior when comparing the cytotoxicity values in both normal and tumor cell lines (mouse L-929 fibroblasts and human hepatocarcinoma cells, respectively) and parasites. On the contrary, MM3 and MM4 appeared to be less toxic than prodigiosins, confirming previous results reported for MM3 by Fiore et al. [27].

The molecules displaying lower toxicity on mammalian cells were less active against *T. cruzi* epimastigotes. Previous observations by Díaz de Greñu et al. demonstrated that the cytotoxicity of these types of molecules was linked to their activity as anionophores [28]. Our results also suggest that the parasiticidal activity of the compounds is linked, at least partly, to their role as anion carriers. Unlike mammalian cells, which contain multiple mitochondria, the mitochondrion in trypanosomatids appears as a single organelle whose proper function is essential for parasite survival, representing an excellent target for anti-infective chemotherapy [36]. Mitochondrial disturbances can be indicated by different means, including modifications in cellular respiration.

Our data showed that natural prodigiosin greatly affected cellular oxygen consumption, as demonstrated by the large decrease in the respiration rates of parasites caused by the compound even at low concentrations (73% at nonlethal concentrations (½ IC_50_)). Our results are consistent with those obtained by Genes et al. [37], who reported strong parasiticidal activity of the pigment, as well as an inhibitory effect on mitochondrial respiration. In fact, the inhibition induced by the pigment was similar to that obtained with thenoyltrifluoroacetone (TTFA), a classical inhibitor of respiratory function, indicating that its biological effect involves the mitochondria. Thus, the most potent respiratory inhibitor was also the most effective trypanocidal compound. This may also be the mechanism of antibacterial activity of prodigiosin against bacteria that rely only on respiration, such as *Pseudomonas aeruginosa* (minimal inhibitory concentration (MIC) of 8 µM), although not the only mechanism since the pigment also kills microorganisms that obtain their energy strictly through fermentation, such as *Enterococcus faecalis* (MIC of 2 µM), as well as facultative bacteria such as *Escherichia coli* (MIC of 2 µM too) [26]. Thus, it seems clear that prodigiosin exerts its antibiotic activity through more than one mechanism.

Furthermore, it is noteworthy that synthetic prodigiosin had a minimal effect on mitochondrial respiration at the concentration equivalent to ½ IC_50,_ but a moderate effect at the highest concentration examined (35.18% of inhibition at ¾ IC_50_). Thus, it was less powerful than natural prodigiosin. This difference between synthetic and natural pigments could have also been due to limitations in the purification protocol, allowing the persistence of other components in the preparations that might also have had some antiparasitic effects.

Regarding the tambjamines, while MM4 had no inhibitory effect on respiration, MM3 elicited respiratory inhibition close to 50% at the maximum concentration measured (¾ IC_50_). These values are similar to those obtained with potassium cyanide (KCN) for respiratory rate inhibition [37], suggesting that MM3 may also act directly on the electron transport chain.

Although it is clear that benznidazole is the most selective compound, its mechanism of toxicity involves the release of highly reactive agents that have potent cytotoxic effects, leading to dermatological, articular, and digestive hypersensitivity reactions, as well as immune thrombocytopenic purpura and other disturbances mediated by antibodies that lead to treatment discontinuation in as much as 20% of cases [38,39,40,41].

Hypersensitivity and immune complexes appear to play important roles in adverse reactions beyond in vitro cytotoxicity. This makes mandatory the replacement or the use of reduced doses of benznidazole, as well as the application of other effective but less toxic drugs.

The toxicity of prodigiosin is well recognized, representing the main limitation of its use as an antimicrobial agent. Despite this, prodigiosin displays a large SI, especially over the intracellular forms of *T. cruzi*, against which prodigiosin had an SI of 50, suggesting its potential as an antichagasic agent. With other approaches, such as the use of nanoformulated preparations, or other pharmaceutical improvements, a dose reduction could be achieved for prodigiosin and other molecules. This would result in a better therapeutic profile. By the moment the bioapplicability of these compounds has been predicted using the web tool SwissADME (Figures S2–S7). Moreover, since they are chemically unrelated to benznidazole, it is highly unlikely that resistance to prodigiosin and the rest of the studied molecules can affect susceptibility to Benznidazole and vice versa.

## Figures and Tables

**Table 1:** The IC_50_ values (µM) of the tested molecules determined in the epimastigotes of *T. cruzi* (CLB strain).

Compound	IC_50_ (µM) ƚ
MM3	4.52 ± 1.43
MM4	5.08 ± 0.62
MM5	129.91 ± 34.66
EH123	198.78 ± 49.93
Obatoclax	3.46 ± 0.96
Synthetic prodigiosin	0.46 ± 0.03
Natural prodigiosin	0.54 ± 0.25
Benznidazole	18.96 ± 7.07

MM3, MM4, MM5, and EH123 are all tambjamines. **ƚ** Results are expressed as the mean IC_50_ value for each compound (± standard deviation) using data obtained in triplicate runs from three independent experiments. The IC_50_ value is the concentration that causes 50% of growth inhibition. This value was calculated using the GraphPad Prism software.

**Table 2:** Biological activity of tambjamines, Obatoclax, synthetic and natural prodigiosins, and benznidazole against trypomastigotes/amastigotes of *T. cruzi* (CLB strain) at the highest concentration assayed (16 µM).

Compound	% Trypomastigote/Amastigote Growth Inhibition (%T/A GI ± SD) *	IC_50_ (µM) ƚ
MM3	97.09 ± 3.01	1.9 ± 0.45
MM4	ND	ND
Obatoclax	88.42 ± 6	2.60 ± 0.4
Synthetic prodigiosin	100 ± 0	0.57 ± 0.19
Natural prodigiosin	100 ± 0	0.1 ± 0.04
Benznidazole	76.01 ± 5.02	1.76 ± 0.34

MM3 and MM4 are tambjamines; SD, standard deviation; ND, not determined. * The results are expressed as the mean value of trypomastigote/amastigote growth inhibition (% T/A GI) at the highest concentration assayed (16 µM) ± SD (n = 9) using data obtained in triplicate runs from three independent experiments. **ƚ** The concentration causing 50% trypomastigote/amastigote growth inhibition was calculated using the GraphPad Prism software.

**Table 3:** FICi values of the combinations between benznidazole and the tested compounds at concentrations equivalent to 0.25x IC_50_.

Combination Drugs	FICi
Benznidazole + 0.25x IC_50_ of MM3	0.79
Benznidazole + 0.25x IC_50_ of MM4	0.70
Benznidazole + 0.25x IC_50_ of Obatoclax	1.80
Benznidazole + 0.25x IC_50_ of synthetic prodigiosin	0.73
Benznidazole + 0.25x IC_50_ of natural prodigiosin	0.81

MM3 and MM4 are tambjamines; FICi, the FIC index.

**Table 4:** Cytotoxicity values of tambjamines, Obatoclax, synthetic and natural prodigiosins, and benznidazole at the concentration of 16 µM in murine fibroblast cells (L-929). IC_50_ were determined testing concentrations of 16, 8, 4, 2, 1, and 0.5 µM for MM3, MM4, Obatoclax and prodigiosins; and concentrations of 256, 128, 64, 32, 16, 8, 4, 2, and 1 µM for EH123; for benznidazole concentrations ranged from 2048 to 16 µM (Figure S1).

	L-929	
Compound	% Cytotoxicity ± SD *	IC_50_ (µM) ƚ
MM3	88.38 ± 2.75	12.21 ± 0.28
MM4	64.24 ± 10.51	14.04 ± 1.28
MM5	ND	ND
EH123	3.72 ± 1.14	171.66 ± 0.5
Obatoclax	63.53 ± 1.63	5.83 ± 1.07
Synthetic prodigiosin	95.75 ± 0.06	3.06 ± 0.01
Natural prodigiosin	95.8 ± 0.09	5 ± 0.83
Benznidazole	0 ± 0	1372.3 ± 0.5

MM3, MM4, MM5, and EH123 are tambjamines; L-929, murine L-929 fibroblasts (NCTC clone 929); SD, standard deviation; ND, not determined. * The results are expressed as the mean of the cytotoxicity values (% cell growth inhibition) at the concentration of 16 µM ± SD (n = 9) using data obtained in triplicate runs from three independent experiments. **ƚ** The concentration causing 50% cell growth inhibition was calculated using the GraphPad Prism software.

**Table 5:** Cytotoxicity values of tambjamines, Obatoclax, synthetic and natural prodigiosins, and benznidazole at the concentrations of 100, 10, and 1 µM in human hepatocarcinoma cells (Hep G2).

Compound	100 µM ± SD (n = 9) *	10 µM ± SD (n = 9) *	1 µM ± SD (n = 9) *	IC_50_ (µM) ƚ
MM3	94.36 ± 0.5	24.87 ± 8.92	0.99 ± 0.92	46.01 ± 8.15
MM4	85.15 ± 9.91	19.07 ± 3.94	0 ± 0	52.54 ± 9.21
Obatoclax	74.8 ± 4.06	63.3 ± 16.95	10.4 ± 4.80	8.18 ± 1.87
Synthetic prodigiosin	95.2 ± 0.94	90.5 ± 0.82	15 ± 6.12	5.15 ± 0.39
Natural prodigiosin	ND	87.97 ± 5.94	2.29 ± 3.97	6.02 ± 0.5
Benznidazole	0.18 ± 0.08	0.44 ± 0.61	0 ± 0	>100

MM3 and MM4 are tambjamines; Hep G2, human hepatocarcinoma cells; SD, standard deviation; ND, not determined. * Data are reported as the mean values of cytotoxicity (% cell growth inhibition) at the concentrations assayed (100, 10, and 1 µM) ± SD (n = 9) using results obtained in triplicate runs from three independent experiments. **ƚ** The concentration causing 50% cell growth inhibition was calculated using the GraphPad Prism software.

**Table 6:** Selectivity index (SI) of tambjamines, Obatoclax, synthetic and natural prodigiosins, and benznidazole against the two forms of *T.cruzi* (CLB strain).

Compound	Epimastigotes	Trypomastigotes
MM3	2.70	6.42
MM4	2.76	ND
EH123	0.86	ND
Obatoclax	1.68	2.24
Synthetic prodigiosin	6.65	5.36
Natural prodigiosin	9.25	50
Benznidazole	72.37	779.71

MM3, MM4, and EH123 are tambjamines; SI, ratio of the IC_50_ value for mouse L-929 fibroblasts to the IC_50_ value for *T. cruzi*; ND, not determined. SI was calculated as follows SI = IC_50_ on mammalian cells/IC_50_ on Parasite.

**Table 7:** Effect of tambjamines, Obatoclax, and synthetic and natural prodigiosins on oxygen uptake in epimastigotes.

	Oxygen Uptake (pmol/sec/Million Cells)	% Oxygen Uptake Inhibition (½ IC_50_) ƚ
Control	2.69 ± 0.06	-
MM3	1.58 ± 0.05	41.26%
MM4	2.62 ± 0.04	2.60%
Natural prodigiosin	0.73 ± 0.06	72.8%
Synthetic prodigiosin	2.33 ± 0.09	13.58%
Obatoclax	2.69 ± 0	0%
	**Oxygen Uptake (pmol/sec/Million Cells)**	**% Oxygen Uptake Inhibition (¾ IC_50_)** **ƚ**
Control	2.69 ± 0.06	-
MM3	1.56 ± 0.06	42%
MM4	2.61 ± 0.04	2.97%
Natural prodigiosin	ND	ND
Synthetic prodigiosin	1.74 ± 0.17	35.18%
Obatoclax	2.69 ± 0	0%

Results are expressed as the mean values of oxygen uptake (pmol/sec/million cells) at the concentration corresponding to ½ IC_50_ ± SD (n = 4). **ƚ** The % oxygen uptake inhibition at the concentration corresponding to ½ IC_50_; ND, not determined. Results are expressed as the mean values of oxygen uptake (pmol/sec/million cells) at the concentration corresponding to **¾** IC_50_ ± SD (n = 4). **ƚ** The % oxygen uptake inhibition at the concentration corresponding to **¾** IC_50._

## Data Availability

Not applicable.

## Supplementary Materials

The following are available online at https://www.mdpi.com/article/10.3390/pharmaceutics13050705/s1, Figure S1: Cytotoxicities of the studied compounds in a mammalian cell line (L-929 fibroblasts), Figure S2: ADME calculated parameters using web tool SwissADME.^1^ for compound **MM3**, Figure S3: ADME calculated parameters using web tool SwissADME.^1^ for compound **MM4**, **Figure S4**: ADME calculated parameters using web tool SwissADME.^1^ for compound **MM5**, Figure S5: ADME calculated parameters using web tool SwissADME.^1^ for compound **EH123**, **Figure S6**: ADME calculated parameters using web tool SwissADME.^1^ for prodigiosin, Figure S7: ADME calculated parameters using web tool SwissADME.^1^ for obatoclax, Table S1: Transmembrane anion transport activity of the compounds expressed as EC50 (µM).
