# Supplementary material for: Tambjamines and Prodiginines: Biocidal Activity against Trypanosoma cruzi
Source: Pharmaceutics. 2021 May 12;13(5):705. doi: 10.3390/pharmaceutics13050705 (PMC8151848; doi:10.3390/pharmaceutics13050705)
Supplement: Supplementary file 1 [file pharmaceutics-13-00705-s001.zip › pharmaceutics-1197866-supplementary.pdf]

pharmaceutics

Supplementary Materials: Tambjamines and Prodiginines: Biocidal activity against *Trypanosoma cruzi*

Rocío Herráez , Roberto Quesada , Norma Dahdah , Miguel Viñas and Teresa Vinuesa

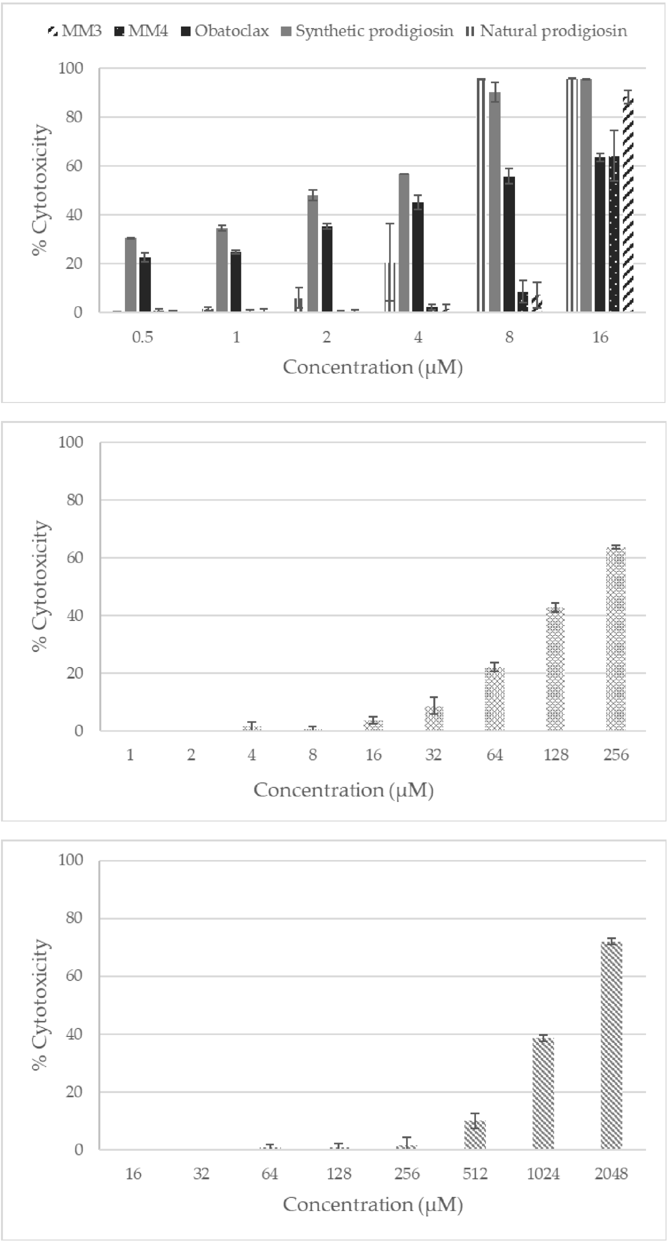

(A)

(B)

(C)

Figure S1. Cytotoxicities of the studied compounds in a mammalian cell line (L-929 fibroblasts). The results are expressed as the mean values of cytotoxicity (% cell growth inhibition) with the corresponding standard deviations. For tambjamines MM3 and MM4; Obatoclax and synthetic and natural prodigiosins **(A),** the assayed concentrations ranged from 0.5 µM to 16 µM. For tambjamine EH123 **(B)**, the assayed concentrations ranged from 1 µM to 256 µM; and for Benznidazole **(C)**, the concentrations ranged from 16 µM to 2048 µM.

Molecule 1

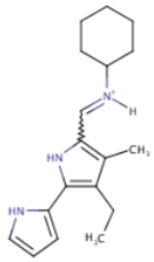

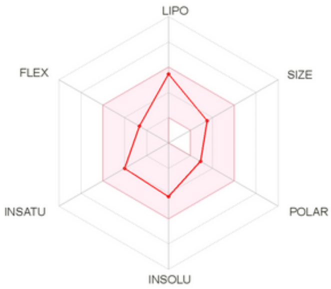

SMILES CCc1c(C)c([nH]c1c1ccc[nH]1)/C=[NH+]/C1CCCCC1

Physicochemical Properties	
Formula	C18H26N3
Molecular weight	284.42 g/mol
Num. heavy atoms	21
Num. arom. heavy atoms	10
Fraction Csp3	0.50
Num. rotatable bonds	4
Num. H-bond acceptors	0
Num. H-bond donors	3
Molar Refractivity	91.37
TPSA	45.55 Å²

Lipophilicity	
Log $P_{o/w}$ (iLOGP)	3.47
Log $P_{o/w}$ (XLOGP3)	4.05
Log $P_{o/w}$ (WLOGP)	2.71
Log $P_{o/w}$ (MLOGP)	2.13
Log $P_{o/w}$ (SILICOS-IT)	5.97
Consensus Log $P_{o/w}$	3.67

Water Solubility	
Log *S* (ESOL)	-4.24
Solubility	1.62e-02 mg/ml ; 5.71e-05 mol/l
Class	Moderately soluble
Log *S* (Ali)	-4.71
Solubility	5.54e-03 mg/ml ; 1.95e-05 mol/l
Class	Moderately soluble
Log *S* (SILICOS-IT)	-6.04
Solubility	2.59e-04 mg/ml ; 9.12e-07 mol/l
Class	Poorly soluble

Pharmacokinetics	
GI absorption	High
BBB permeant	Yes
P-gp substrate	No
CYP1A2 inhibitor	Yes
CYP2C19 inhibitor	Yes
CYP2C9 inhibitor	No
CYP2D6 inhibitor	Yes
CYP3A4 inhibitor	No
Log K_p (skin permeation)	-5.16 cm/s

Druglikeness	
Lipinski	Yes; 0 violation
Ghose	Yes
Veber	Yes
Egan	Yes
Muegge	Yes
Bioavailability Score	0.55

Medicinal Chemistry	
PAINS	0 alert
Brenk	1 alert: imine_1
Leadlikeness	No; 1 violation: XLOGP3>3.5
Synthetic accessibility	3.41

Figure S2. ADME calculated parameters using web tool SwissADME.[1] for compound **MM3.**

Molecule 2

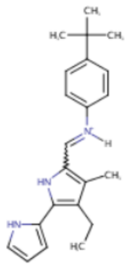

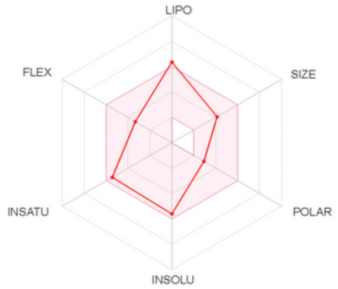

SMILES CCc1c(C)c([nH]c1c1ccc[nH]1)/C=[NH+]/c1ccc(cc1)C(C)(C)C

Physicochemical Properties	
Formula	C22H28N3
Molecular weight	334.48 g/mol
Num. heavy atoms	25
Num. arom. heavy atoms	16
Fraction Csp3	0.32
Num. rotatable bonds	5
Num. H-bond acceptors	0
Num. H-bond donors	3
Molar Refractivity	108.95
TPSA	45.55 Å²

Lipophilicity	
Log $P_{o/w}$ (iLOGP)	3.95
Log $P_{o/w}$ (XLOGP3)	5.66
Log $P_{o/w}$ (WLOGP)	4.01
Log $P_{o/w}$ (MLOGP)	3.06
Log $P_{o/w}$ (SILICOS-IT)	7.22
Consensus Log $P_{o/w}$	4.78

Water Solubility	
Log *S* (ESOL)	-5.62
Solubility	7.97e-04 mg/ml ; 2.38e-06 mol/l
Class	Moderately soluble
Log *S* (Ali)	-6.38
Solubility	1.39e-04 mg/ml ; 4.16e-07 mol/l
Class	Poorly soluble
Log *S* (SILICOS-IT)	-8.31
Solubility	1.65e-06 mg/ml ; 4.94e-09 mol/l
Class	Poorly soluble

Pharmacokinetics	
GI absorption	High
BBB permeant	Yes
P-gp substrate	No
CYP1A2 inhibitor	Yes
CYP2C19 inhibitor	Yes
CYP2C9 inhibitor	No
CYP2D6 inhibitor	No
CYP3A4 inhibitor	Yes
Log K_p (skin permeation)	-4.32 cm/s

Druglikeness	
Lipinski	Yes; 0 violation
Ghose	Yes
Veber	Yes
Egan	Yes
Muegge	No; 1 violation: XLOGP3>5
Bioavailability Score	0.55

Medicinal Chemistry	
PAINS	0 alert
Brenk	1 alert: imine_1
Leadlikeness	No; 1 violation: XLOGP3>3.5
Synthetic accessibility	3.69

Figure S3. ADME calculated parameters using web tool SwissADME.[1] for compound **MM4.**

Molecule 3

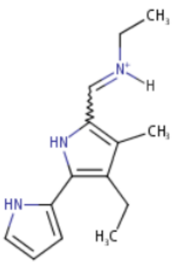

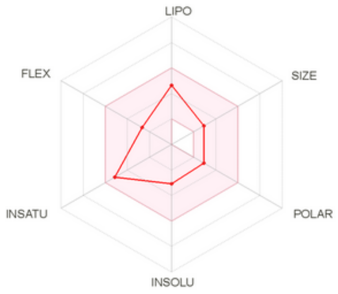

SMILES CCc1c(C)c([nH]c1c1ccc[nH]1)/C=[NH+]/CC

Physicochemical Properties	
Formula	C14H20N3
Molecular weight	230.33 g/mol
Num. heavy atoms	17
Num. arom. heavy atoms	10
Fraction Csp3	0.36
Num. rotatable bonds	4
Num. H-bond acceptors	0
Num. H-bond donors	3
Molar Refractivity	74.25
TPSA	45.55 Å²

Lipophilicity	
Log $P_{o/w}$ (iLOGP)	2.85
Log $P_{o/w}$ (XLOGP3)	2.63
Log $P_{o/w}$ (WLOGP)	1.40
Log $P_{o/w}$ (MLOGP)	1.14
Log $P_{o/w}$ (SILICOS-IT)	5.09
Consensus Log $P_{o/w}$	2.62

Water Solubility	
Log *S* (ESOL)	-3.10
Solubility	1.85e-01 mg/ml ; 8.01e-04 mol/l
Class	Soluble
Log *S* (Ali)	-3.24
Solubility	1.33e-01 mg/ml ; 5.80e-04 mol/l
Class	Soluble
Log *S* (SILICOS-IT)	-5.41
Solubility	9.02e-04 mg/ml ; 3.92e-06 mol/l
Class	Moderately soluble

Pharmacokinetics	
GI absorption	High
BBB permeant	Yes
P-gp substrate	No
CYP1A2 inhibitor	Yes
CYP2C19 inhibitor	Yes
CYP2C9 inhibitor	No
CYP2D6 inhibitor	Yes
CYP3A4 inhibitor	No
Log K_p (skin permeation)	-5.84 cm/s

Druglikeness	
Lipinski	Yes; 0 violation
Ghose	Yes
Veber	Yes
Egan	Yes
Muegge	Yes
Bioavailability Score	0.55

Medicinal Chemistry	
PAINS	0 alert
Brenk	1 alert: imine_1
Leadlikeness	No; 1 violation: MW<250
Synthetic accessibility	3.14

Figure S4. ADME calculated parameters using web tool SwissADME.[1] for compound **MM5.**

Molecule 4

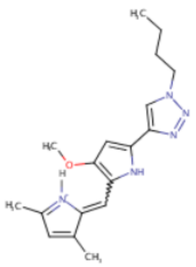

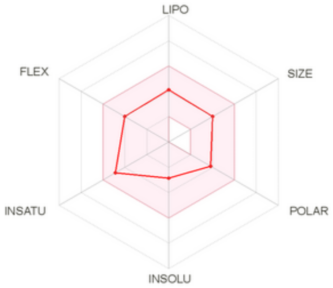

SMILES CCCCn1nnc(c1)c1cc(c([nH]1)/C=C/1\[NH+]=C(C=C1C)C)OC

Physicochemical Properties	
Formula	C18H24N5O
Molecular weight	326.42 g/mol
Num. heavy atoms	24
Num. arom. heavy atoms	10
Fraction Csp3	0.39
Num. rotatable bonds	6
Num. H-bond acceptors	3
Num. H-bond donors	2
Molar Refractivity	101.50
TPSA	69.70 Å²

Lipophilicity	
Log $P_{o/w}$ (iLOGP)	3.58
Log $P_{o/w}$ (XLOGP3)	1.70
Log $P_{o/w}$ (WLOGP)	1.43
Log $P_{o/w}$ (MLOGP)	1.37
Log $P_{o/w}$ (SILICOS-IT)	4.26
Consensus Log $P_{o/w}$	2.47

Water Solubility	
Log *S* (ESOL)	-2.85
Solubility	4.64e-01 mg/ml ; 1.42e-03 mol/l
Class	Soluble
Log *S* (Ali)	-2.78
Solubility	5.43e-01 mg/ml ; 1.66e-03 mol/l
Class	Soluble
Log *S* (SILICOS-IT)	-5.17
Solubility	2.22e-03 mg/ml ; 6.79e-06 mol/l
Class	Moderately soluble

Pharmacokinetics	
GI absorption	High
BBB permeant	Yes
P-gp substrate	Yes
CYP1A2 inhibitor	Yes
CYP2C19 inhibitor	Yes
CYP2C9 inhibitor	No
CYP2D6 inhibitor	No
CYP3A4 inhibitor	No
Log K_p (skin permeation)	-7.08 cm/s

Druglikeness	
Lipinski	Yes; 0 violation
Ghose	Yes
Veber	Yes
Egan	Yes
Muegge	Yes
Bioavailability Score	0.55

Medicinal Chemistry	
PAINS	0 alert
Brenk	0 alert
Leadlikeness	Yes
Synthetic accessibility	4.33

Figure S5. ADME calculated parameters using web tool SwissADME.[1] for compound **EH123.**

Molecule 5

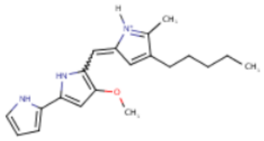

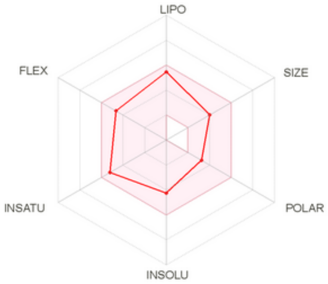

SMILES CCCCCC1=C/C(=C/c2[nH]c(cc2OC)c2ccc[nH]2)/[NH+]=C1C

Physicochemical Properties	
Formula	C20H26N3O
Molecular weight	324.44 g/mol
Num. heavy atoms	24
Num. arom. heavy atoms	10
Fraction Csp3	0.35
Num. rotatable bonds	7
Num. H-bond acceptors	1
Num. H-bond donors	3
Molar Refractivity	105.81
TPSA	54.78 Å²

Lipophilicity	
Log $P_{o/w}$ (iLOGP)	3.73
Log $P_{o/w}$ (XLOGP3)	4.06
Log $P_{o/w}$ (WLOGP)	2.93
Log $P_{o/w}$ (MLOGP)	1.85
Log $P_{o/w}$ (SILICOS-IT)	6.29
Consensus Log $P_{o/w}$	3.77

Water Solubility	
Log *S* (ESOL)	-4.26
Solubility	1.80e-02 mg/ml ; 5.55e-05 mol/l
Class	Moderately soluble
Log *S* (Ali)	-4.91
Solubility	3.95e-03 mg/ml ; 1.22e-05 mol/l
Class	Moderately soluble
Log *S* (SILICOS-IT)	-6.76
Solubility	5.68e-05 mg/ml ; 1.75e-07 mol/l
Class	Poorly soluble

Pharmacokinetics	
GI absorption	High
BBB permeant	Yes
P-gp substrate	Yes
CYP1A2 inhibitor	Yes
CYP2C19 inhibitor	Yes
CYP2C9 inhibitor	No
CYP2D6 inhibitor	No
CYP3A4 inhibitor	Yes
Log K_p (skin permeation)	-5.40 cm/s

Druglikeness	
Lipinski	Yes; 0 violation
Ghose	Yes
Veber	Yes
Egan	Yes
Muegge	Yes
Bioavailability Score	0.55

Medicinal Chemistry	
PAINS	0 alert
Brenk	0 alert
Leadlikeness	No; 1 violation: XLOGP3>3.5
Synthetic accessibility	4.28

Figure S6. ADME calculated parameters using web tool SwissADME.[1] for prodigiosin.

Molecule 6

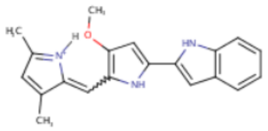

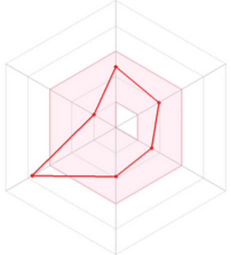

SMILES COc1cc([nH]c1/C=C/1\[NH+]=C(C=C1C)C)c1cc2c([nH]1)cccc2

Physicochemical Properties	
Formula	C20H20N3O
Molecular weight	318.39 g/mol
Num. heavy atoms	24
Num. arom. heavy atoms	14
Fraction Csp3	0.15
Num. rotatable bonds	3
Num. H-bond acceptors	1
Num. H-bond donors	3
Molar Refractivity	104.09
TPSA	54.78 Å²

Lipophilicity	
Log $P_{o/w}$ (iLOGP)	3.23
Log $P_{o/w}$ (XLOGP3)	2.88
Log $P_{o/w}$ (WLOGP)	2.52
Log $P_{o/w}$ (MLOGP)	1.93
Log $P_{o/w}$ (SILICOS-IT)	5.80
Consensus Log $P_{o/w}$	3.27

Water Solubility	
Log *S* (ESOL)	-3.86
Solubility	4.37e-02 mg/ml ; 1.37e-04 mol/l
Class	Soluble
Log *S* (Ali)	-3.69
Solubility	6.50e-02 mg/ml ; 2.04e-04 mol/l
Class	Soluble
Log *S* (SILICOS-IT)	-6.82
Solubility	4.85e-05 mg/ml ; 1.52e-07 mol/l
Class	Poorly soluble

Pharmacokinetics	
GI absorption	High
BBB permeant	Yes
P-gp substrate	Yes
CYP1A2 inhibitor	Yes
CYP2C19 inhibitor	Yes
CYP2C9 inhibitor	No
CYP2D6 inhibitor	Yes
CYP3A4 inhibitor	Yes
Log K_p (skin permeation)	-6.20 cm/s

Druglikeness	
Lipinski	Yes; 0 violation
Ghose	Yes
Veber	Yes
Egan	Yes
Muegge	Yes
Bioavailability Score	0.55

Medicinal Chemistry	
PAINS	0 alert
Brenk	0 alert
Leadlikeness	Yes
Synthetic accessibility	4.00

Figure S7. ADME calculated parameters using web tool SwissADME.[1] for obatoclax.

[1] Daina, A., Michielin, O. & Zoete, V. SwissADME: a free web tool to evaluate pharmacokinetics, drug-likeness and medicinal chemistry friendliness of small molecules. Sci Rep 7, 42717 (2017). https://doi.org/10.1038/srep42717

Table S1. Transmembrane anion transport activity of the compounds expressed as EC50 (μM) [a] Values from reference [1]; [b] The compound was not active enough to calculate EC_{50} value. [c] Values from reference [2]; [d] Values from reference [3]; ND not determined.

Compound	EC50 (μM) NO3–/Cl–	EC50 (μM) HCO3–/Cl–
**MM3**	0.035 [a]	ND
**MM4**	ND	ND
**MM5**	- [b]	- [b]
**EH123**	0.024 [c] c	1.911 [c]
**Prodigiosin**	0.002	0.06
**Obatoclax**	0.010	0.28[d]

[1] Hernando, E.; Capurro, V.; Cossu, C.; Fiore, M.; García-Valverde, M.; Soto-Cerrato, V.; Pérez-Tomás, R.; Moran, O.; Zegarra-Moran O. and Quesada R. Small molecule anionophores promote transmembrane anion permeation matching CFTR activity. *Sci. Rep.*, **2018**, 8, 2608.

[2] Ambra Gianotti, A.; Capurro, V.; Delpiano, L.; Mielczarek, M.; García-Valverde M.; Carreira-Barral, I.; Ludovico, A.; Fiore, M.; Baroni, D.; Moran, O.; Quesada R. and Caci, E. Small molecule anion carriers correct abnormal airway surface liquid properties in cystic fibrosis airway epithelia. *Int. J. Mol. Sci.*, **2020**, 21(4):1488.

[3] Díaz de Greñu, B.; Iglesias Hernández, P.; Espona, M.; Quiñonero, D.; Light, M.E.; Torroba, T.; *et al*. Synthetic prodiginine Obatoclax (GX15-070) and related analogues: anion binding, transmembrane transport, and cytotoxicity properties. *Chem Eur J*. **2011**, *17(50)*,14074-14083.
